# Statistical Analysis of Microarray Data with Replicated Spots: A Case Study with *Synechococcus* WH8102

**DOI:** 10.1155/2009/950171

**Published:** 2009-04-23

**Authors:** E. V. Thomas, K. H. Phillippy, B. Brahamsha, D. M. Haaland, J. A. Timlin, L. D. H. Elbourne, B. Palenik, I. T. Paulsen

**Affiliations:** ^1^Department of Independent Surveillance Assessment and Statistics, Sandia National Laboratories, Albuquerque, NM 87185-0829, USA; ^2^National Center for Biotechnology Information, National Library of Medicine, National Institute of Health, Bethesda, MD 20894, USA; ^3^Scripps Institution of Oceanography, University of California at San Diego, La Jolla, CA 92093-0202, USA; ^4^Department of Biomolecular Analysis and Imaging, Sandia National Laboratories, Albuquerque, NM 87185-0895, USA; ^5^Department of Chemistry and Biomolecular Sciences, Macquarie University, Sydney, NSW 2109, Australia

## Abstract

Until recently microarray experiments often involved relatively few arrays with only a single representation of each gene on each array. A complete genome microarray with multiple spots per gene (spread out spatially across the array) was developed in order to compare the gene expression of a marine cyanobacterium and a knockout mutant strain in a defined artificial seawater medium. Statistical methods were developed for analysis in the special situation of this case study where there is gene replication within an array and where relatively few arrays are used, which can be the case with current array technology. Due in part to the replication within an array, it was possible to detect very small changes in the levels of expression between the wild type and mutant strains. One interesting biological outcome of this experiment is the indication of the extent to which the phosphorus regulatory system of this cyanobacterium affects the expression of multiple genes beyond those strictly involved in phosphorus acquisition.

## 1. Introduction

Microarray experiments provide high-throughput gene expression data required for elucidating networks and pathways occurring in organisms and for validating models derived from other experimental data. The quality of models and inference derived from microarray experiments obviously depends on the quality of the microarray data. For example, predictive models are hard to develop or validate if microarray data have high false positive and/or false negative rates for identifying differential gene expression. Thus, it is important to make results from microarray experiments as reproducible and reliable as possible. In addition, it is important to institute a process to monitor, assess, and ultimately improve the quality of the microarray data. 

A number of researchers have identified a variety of sources of variation which affect the reproducibility of microarray data. Statistically designed microarray experiments that include replication have been critical to understanding, assessing, and improving the quality of microarray data [1–3]. In our own experience, through various statistically designed experiments, we have been able to identify and correct problems with the training of operators (scanner), inhomogeneous hybridizations, inadequate blocking of the poly-L-lysine coatings, print problems, and normalization procedures. 

Along with others (see, e.g., [4, 5]), we have often observed effects of sources of variation that are manifested spatially. Frequently, these effects are most striking from the top to the bottom of an array. We have reduced these effects by modifying our hybridization processes to include a gentle rocking of the hybridization chamber (e.g., see also [6]). Nevertheless, even after this process modification, we have observed spatial effects that can result in apparent differences in relative expression of 30% or more across an array. Variation of this magnitude can be problematic when one is trying to identify genes that are weakly up- or downregulated. Thus, it is important to be able to easily monitor spatial effects. 

The continuing effects of spatially-related sources of variation (including instances where printing or hybridization artifacts render a portion of an array completely unusable) have motivated the development of print designs that include replicate spots per gene that are spatially distributed over the array and printed with different pins. Combining this approach along with multiple technical and biological replicates is an effective way to provide the necessary data to enable a meaningful analysis that is able to separate the effects of multiple sources of variation and produce a more accurate assessment of a gene's true expression level.

In our study of gene expression in *Synechococcus* WH8102, we have constructed a complete genome microarray with multiple spots per gene spread out spatially across the array. This microarray is being used as a platform to compare various regulatory mutants of *Synechococcus* with the wild type under a variety of conditions and to study the effects of different sources of nitrogen or phosphorus for growth of the wild type [7]. Here we report a case study of the analysis of one of these experiments, comparing phosphorus metabolism of wild type and a strain in which a phosphorus-related response regulator gene has been inactivated. 

Phosphorus can sometimes be a limiting nutrient in marine ecosystems (see, e.g., [8]). The availability of intracellular phosphorus for growth and the response of the cell to changing phosphorus levels are controlled in many bacteria by a two-component system including a histidine kinase (sensor) and response regulator (DNA-binding protein) pair, *PhoR* and *PhoB*, respectively, [9, 10]. In *Synechococcus* WH8102 the gene SYNW0947 is a *PhoB* homologue [11]. This gene was insertionally inactivated using the methods described in [12]. Gene expression of this mutant was then compared to that of wild type grown under standard conditions. This comparison along with other studies of cells grown under different phosphorus conditions will lead to an understanding of the phosphate regulon of these ecologically important microorganisms. 

## 2. Materials and Methods

### 2.1. Experimental

The complete genome microarray for *Synechococcus* sp. strain WH8102 was used as the platform for a replicated dye-swap design [13] involving four slides (see Table 1). A single sample of the wild-type *Synechococcus* (WH8102) RNA was used as a control, while two samples of the mutant RNA were obtained for comparison. The microarray consists of a mixed population of PCR amplicons (2142 genes) and 70-mer oligonucleotides (389 genes). Unique PCR amplicons representing each gene are approximately 800 bp in size or smaller if the gene size is smaller. Unique 70-mer oligonucleotides were utilized for genes under 300 bp in size and for the two genes that we were unable to amplify by PCR. Six complete replicates of the 2531-member gene set were printed on aminosilane coated Corning ultraGAP glass slides using an Intelligent Automation Systems (IAS) high-precision microarray-printing robot with 48 pins for printing and irreversibly bound by UV-crosslinking at 250 mJ. Each array slide also includes a variety of negative controls (50% DMSO/50% deionized water) and positive controls (including a total mix of WH8102 PCR amplicons, spiked *Arabidopsis* PCR amplicons and 70-mer oligonucleotides).

The amplicons/oligonucleotides were split into two separate sets of 384-well plates with each amplicon/oligonucleotide in a different well position. This enabled us to develop a print pattern with each of the six replicate spots located in different blocks separated both horizontally and vertically across the slide.

The * Synechococcus* strains were grown in standard ocean water (SOW) medium, and total RNA was extracted using a Trizol-based method (Invitrogen) following manufacturers recommendations and purified using a mini RNeasy kit (Qiagen). The purity and yield of the RNA were determined spectrophotometrically by measuring optical density at wavelengths of 260 and 280 nm. An indirect labeling method was used to label cDNA, where cDNA was synthesized in the presence of a nucleoside triphosphate analog containing a reactive aminoallyl group to which the fluorescent dye molecule was coupled. Prior to hybridization, labeled cDNA was scanned spectrophotometrically to ensure optimal dye incorporation per sample for adequate signal intensity. A single sample of the wild-type *Synechococcus* (WH8102) RNA was used as a control, while two samples of the mutant RNA were obtained for comparison. Hybridizations were performed as previously described in [14], and slides were promptly scanned at a 10-*μ*m resolution using an Axon 4000B scanner with GenePix 4.0 software.

**Figure 1:**
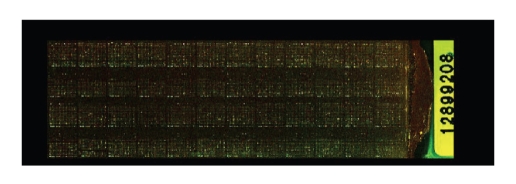
Full genome *Synechococcus* array.

**Figure 2:**
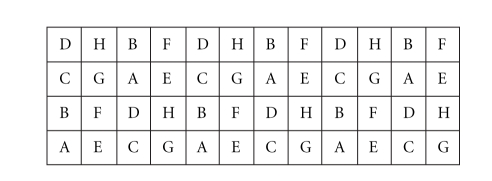
Full genome *Synechococcus* array (showing block positions of replicates).

Figure 1 displays the fluorescence image of a hybridized array. The array contains 19 200 spots in 48 blocks with 20 rows and columns in each block. Each of the genes appears in six different blocks within the array (and therefore is printed by six of the 48 different pins) and is assigned to a letter {A, B, C, D, E, F, G, or H}. For a given gene, the block positions are given by the position of its assigned letter in Figure 2. The position of a given gene within a block is consistent across its six replicates. In addition, the array contains a number of control spots, both positive and negative. Some control spots are used for alignment (e.g., see first column of the first few rows of each block), and others are used for quality control.

### 2.2. Data Preprocessing

TIGR's SPOTFINDER and MIDAS software [15] was used to process the four microarray images. This processing resulted nominally in a “4arrays × 6gene replicates × 2531genes” data array consisting of the relative intensities, *I*_Treatment_/*I*_Control_, of each spot. The relatively few spots that were rejected were rejected only on the basis of poor visual quality. Spots with low intensity were not automatically rejected, resulting in quantitative representation of a vast majority of the genes over six spatially varying replicate spots on each array. 

We use log_2_(*I*_Treatment_/*I*_Control_) as a basis for the quantitative analysis that follows. 

### 2.3. Array Normalization

A two-step modeling process analogous to the approach used in [16] was used to normalize the data. However, unlike in [16], log(ratios) were used rather than log(intensities). First, the data were normalized by subtracting the slide-specific global average log-ratio. This adjusted for global effects (across all spots on a slide) due to the dye configuration (standard versus flipped) and/or the biological replicate. To formalize this, let *Y*_*gij*_ be the observed log-ratio associated with the *g*th gene, *i*th biological replicate, and *j*th * dye* configuration (*i* = 1:2 and *j* = 1:2). Then, the normalized expression data are given by $R_{gij}=Y_{gij}-{\overset{¯}{Y}}_{.ij}$, where ${\bar{Y}}_{.ij}$ represents the average expression level of the slide corresponding to the *i*th biological replicate and the *j*th * dye* configuration. 

### 2.4. Variance Components Analysis

Following array normalization, a variance components analysis was used to partition the observed variability in expression level across replicate arrays. The purpose of this analysis was to help further understanding the relative magnitudes of the various sources of experimental variation. A model for the normalized expression data is given by *R*_*gij*_ = *G*_*g*_ + (*BG*)_*gi*_ + (*DG*)_*gj*_ + *ε*_*gij*_, where *R*_*gij*_ is the observed normalized relative expression of the *g*th gene for the *i*th biological replicate and the *j*th *dye* configuration. *G*_*g*_ represents the true (but unknown) relative expression level of the *g*th gene, and (*BG*)_*gi*_ and (*DG*)_*gj*_ represent the random gene-specific effects associated with the biological replicate and the dye. The term *ε*_*gij*_ is representative of a nonspecific random effect that is unrelated to the biological replicate or the dye. The variances of these random effects are given by *σ*_*b*_^2^, *σ*_*d*_^2^, and *σ*_*ε*_^2^. The true expression level of a given gene is estimated as the average value of *R* over the four slides: ${\hat{G}}_{g}=(1/4)\cdot \sum_{i=1}^{2}\sum_{j=1}^{2}R_{gij}.$

One degree-of-freedom estimates for the three variance components can be obtained for *each gene* via an analysis of variance (ANOVA) of the values of *R* (see, e.g., [17]):

(1) σ^ε2=∑i=12 ∑j=12(Rgij−R¯gi⋅−R¯g⋅j+G^g)2,σ^b2=max(0,∑i=12(R¯gi⋅−G^g)2−12⋅σ^ε2),σ^d2=max(0,∑j=12(R¯g⋅j−G^g)2−12⋅σ^ε2),

where

(2) $${\overset{¯}{R}}_{gi\cdot }=\frac{1}{2}\cdot \overset{2}{\underset{j=1}{\sum }}\;R_{gij},\;{\overset{¯}{R}}_{g\cdot j}=\frac{1}{2}\cdot \overset{2}{\underset{i=1}{\sum }}\;R_{gij}.$$

Smoothed versions (“running 10%-trimmed means”) of these summary statistics were also computed. That is, for each case, $\left(\hat{G},\hat{\sigma }\right)$ are ordered by the value of $\hat{G}$, resulting in $\left\{\hat{G}\left(1\right),\hat{G}\left(2\right),\ldots ,\hat{G}\left(N\right)\right\}$ and $\left\{\hat{\sigma }\left(1\right),\hat{\sigma }\left(2\right),\ldots ,\hat{\sigma }\left(N\right)\right\}$, where *N* is the number of genes considered. The left endpoint of each curve is given by the co-ordinates: ${\text{median}}_{i=1:100}\left(\hat{G}\left(i\right)\right)$ and trimmed meani=1:100(σ^2(i)). In general the *j*th point of each curve is given by the coordinates: ${\text{median}}_{i=j:100+j-1}\left(\hat{G}\left(i\right)\right)$ and trimmed meani=j:100+j-1(σ^2(i)). The trimmed mean is the average of the 100 observations with the smallest and largest 5 observations removed. In contrast to the noisy individual values of ${\hat{\sigma }}_{d},{\hat{\sigma }}_{b},$ and ${\hat{\sigma }}_{\varepsilon }$ (which are each associated with a single degree of freedom), these curves provide a smooth visual perspective regarding the behavior of each of the variance components with varying levels of $\hat{G}$. In addition, statistics derived from these curves are used as a basis for making inference. 

### 2.5. Standard Error of ${\hat{G}}_{g}$

Based on the gene-specific variance components estimates, a direct (but noisy) estimate of the standard error of ${\hat{G}}_{g}$ is given by

(3) σ^G^g=σ^d2(G^g)2+σ^b2(G^g)2+σ^ε2(G^g)4.

Alternatively, we can assume that the smooth versions of these variance components are more representative of the underlying true levels of the variance components and that these variance components are dependent only on the level of *G*_*g*_. Denote these smooth curves by σ˜d(G^), σ˜b(G^), and σ˜ε(G^). Based on these smooth curves, the estimated standard error of $\hat{G}$ is given by

(4) σ˜G^=σ˜d2(G^)2+σ˜b2(G^)2+σ˜ε2(G^)4.

We are most interested in the constituent variance components and overall level of variability of $\hat{G}$ when *G* = 0 (corresponding to the case when the hypothetical treatment gene expression level is unchanged from the control). In practice, since we do not know what the true gene expression level (*G*) is, we are interested in the level of variability when $\hat{G}\approx 0$ (corresponding to the case where there is a relatively little observed change in the gene expression level). Evaluating σ˜G^ at $\hat{G}=0$, we computed

(5) $${\overset{˜}{\sigma }}_{0}=\sqrt{\frac{{\overset{˜}{\sigma }}_{d}^{2}\left(0\right)}{2}+\frac{{\overset{˜}{\sigma }}_{b}^{2}\left(0\right)}{2}+\frac{{\overset{˜}{\sigma }}_{\varepsilon }^{2}\left(0\right)}{4}.}$$

### 2.6. Test Statistic

A test statistic was developed to form the basis for our assessment of whether a particular gene was significantly upregulated or downregulated. The test statistic is Sg=G^g/σ^G^g(com), where σ^G^g(com)=max (σ^G^g,σ˜0). The purpose of this *combined* estimate for the standard error of ${\hat{G}}_{g}$ is to prevent the computed statistic, *S*_*g*_, from being too large (in absolute value) based on a chance small value of σ^G^gthat is not representative of the true value of σG^g. Such nonrepresentative small values of σ^G^g would not be uncommon due to the small sample size of 4 arrays. Note that Cui and Churchill [18] discuss other modified *t*-tests used to assess differential expression. The floor of σ^G^g(com), ${\tilde{\sigma }}_{0}$, is analogous to the “fudge” term used in the widely used significance analysis of microarrays method (SAM) that was developed by Tusher et al. [19]. The distribution of this test statistic, when *G*_*g*_ = 0, is complicated and depends on assumptions about the random effects in the normalized gene expression model: *R*_*gij*_ = *G*_*g*_ + (*BG*)_*gi*_ + (*DG*)_*gj*_ + *ε*_*gij*_.

If we assume that the random effects are normally distributed with zero mean and specified variances (*σ*_*d*_^2^, *σ*_*b*_^2^, *σ*_*ε*_^2^), then selected percentiles of the null distribution of the test statistic can be estimated by simulating gene expression data via the model: *R*_*ij*_ = *G* + *B*_*i*_ + *D*_*j*_ + *ε*_*ij*_ (*i* = 1:2 and *j* = 1:2) with *G* = 0. The simulation is set up to mimic the actual experiment: a replicated dye-swap design involving four slides and two biological samples. The experiment can be simulated many times with each realization resulting in a value for the test statistic, *S*_*g*_. Selected order statistics from the distribution of *S*_*g*_ values obtained from the simulations provide approximate percentiles of the null distribution. 

## 3. Results and Discussion

### 3.1. Assessment of Slide Quality and Identification of Anomalous Data

**Figure 3:**
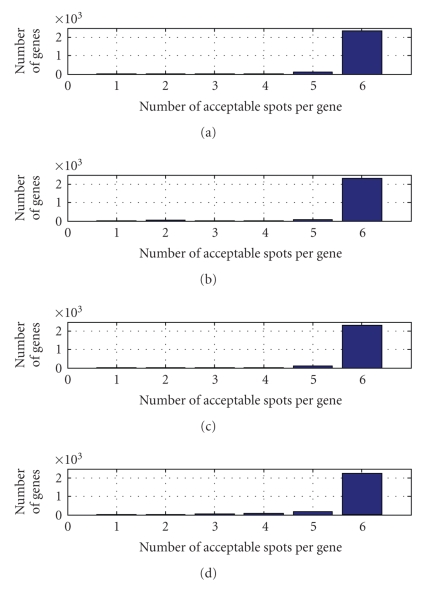
Number of genes with {1, 2, 3, 4, 5, or 6} acceptable spots per slide. Slides 1, 2, 3, and 4 are represented from top to bottom.

The four microarray images each containing six replicate representations of the 2531 genes were processed into a 4 × 6 × 2531 data array of relative intensities. Spots were rejected solely on the basis of poor quality resulting in quantitative representation of a vast majority of the genes over six spatially varying replicate spots on each array. Figure 3 illustrates the distribution of acceptable spots per gene on each array. We recommend a graphic of this nature for experiments which have multiple spots per gene printed on each slide as it allows for a quick assessment of the relative quality of each slide in the study.

**Figure 4:**
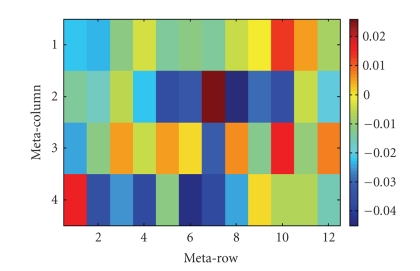
Median log-ratios within each block: slide no. 1.

Here, due to the nature of the print design it is also possible to examine whether there are gross spatial effects within each slide. Note that the 48 blocks are arranged in a 12 meta-row by 4 meta-column configuration. About 300 genes are printed in each block. Figure 4 displays the median log-ratios of spots within each block for slide no. 1. Assuming that the typical gene is not differentially expressed, we expect that the median log-ratio for each block to be close to zero. Overall, the median log-ratios of slide no. 1 are slightly negative, but quite small in magnitude (effects span about 0.07 log _2_ units). However, as is the case with the other slides, no large block-to-block spatial effects are observed. Note that this is in contrast to earlier *Synechococcus* experiments that we conducted in which much larger spatial effects (spanning about 0.3 log_2_ units across slides) were observed but later improved by changing hybridization conditions. If such large effects were present in association with a traditional print design, the perceived expression level of genes with spots located only in the discrepant area would be inaccurate. In our print design, the influence of the spatial effects is minimized since affected genes are represented elsewhere in spatially distinct locations on the slide.

**Figure 5:**
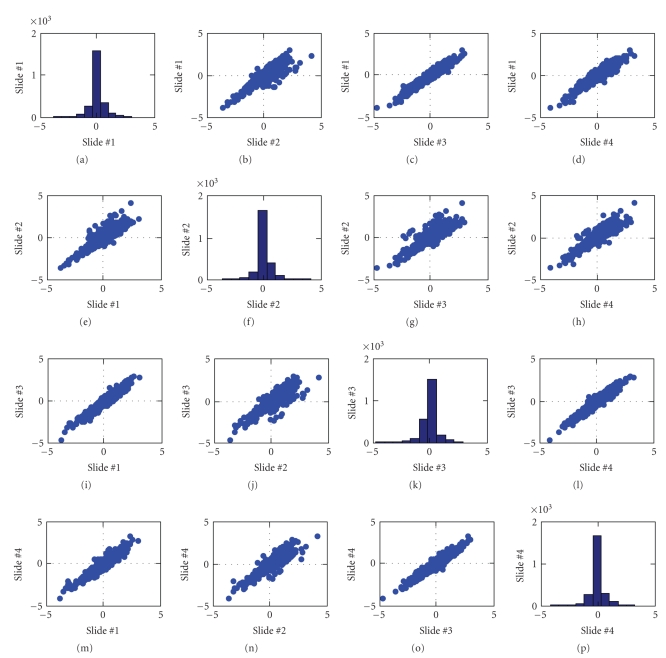
Scatterplot matrix of the median log-ratios. The expression distribution of each slide is represented along the diagonal of the scatterplot matrix.

**Figure 6:**
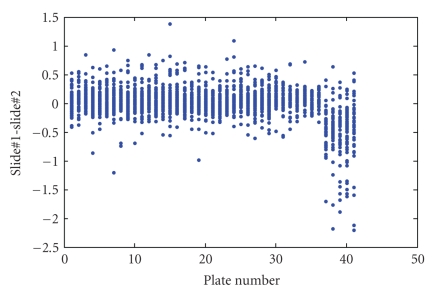
Difference in median log-ratios (slide no. 1-slide no. 2) versus plate number.

**Figure 7:**
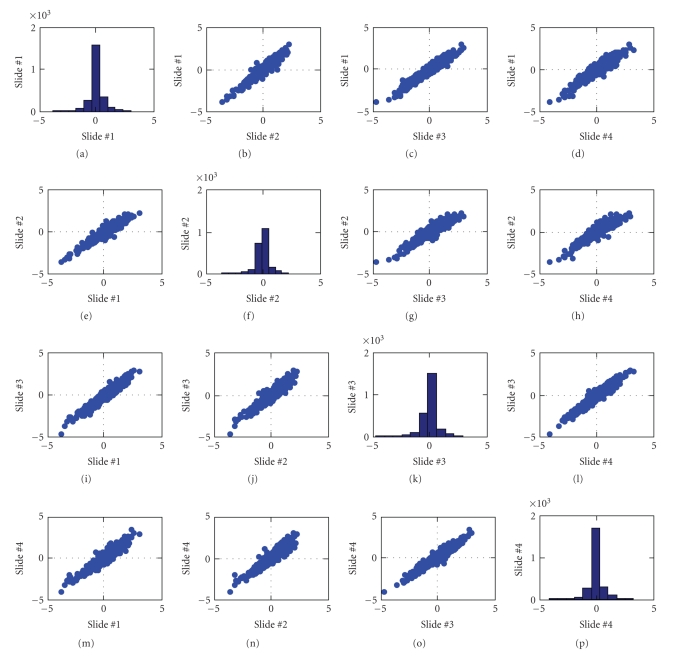
Scatterplot matrix of the median log-ratios (genes from 5 suspect plates removed). The expression distribution of each slide is represented along the diagonal of the scatterplot matrix.

The results from the 2408 genes represented by at least 4 spots on each array of “acceptable” quality form the basis for further analysis and modeling. For each of these genes, we computed median(log_2_(*I*_Treatment_/*I*_Control_)) across the acceptable replicate spots within each slide. Figure 5 presents the relationship between values of median log-ratios across the four slides. For the most part, the median log-ratios are quite consistent across the four slides. However, there are a number of genes that produced atypically large log-ratios for slide no. 2 (see scatter plots in the second row and the second column of Figure 5). A graphical analysis comparing slide no. 1 to slide no. 2 shows that these genes were associated with the last five print plates in the print run (see Figure 6). Although not confirmed, it is suspected that these effects are due to evaporation of the print solution. Figure 7 presents the relationship between values of median log-ratios across the four slides after excluding the 271 genes associated with the five suspect print plates.

### 3.2. Results of Array Normalization

**Figure 8:**
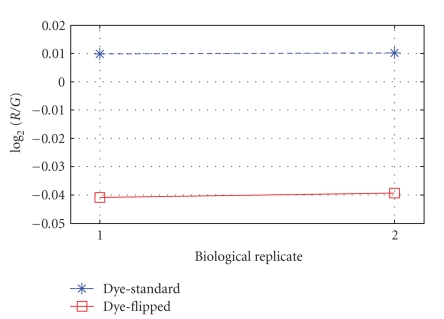
Average effects of dye and biological replicate (genes from suspect plates removed).

The remaining data (involving 2137 genes) were normalized using the procedure described in Section 2.3. Figure 8 displays the values of ${\overset{¯}{Y}}_{.ij}$ and hence illustrates the average effects of dye and biological replicate over the 4 slides. Notice that across a slide the average effect of the dye is about 0.05 log_2_ units, while the average effect due to the biological replicate is barely perceptible.

### 3.3. Results of Variance Components Analysis

**Figure 9:**
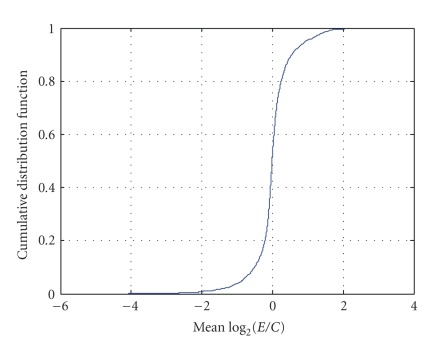
Cumulative distribution of estimated gene expression levels (G^g).

**Figure 10:**
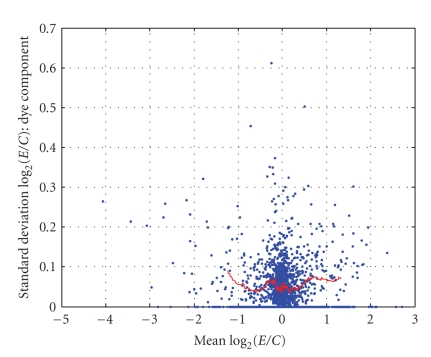
σ^d2 versus G^g.

**Figure 11:**
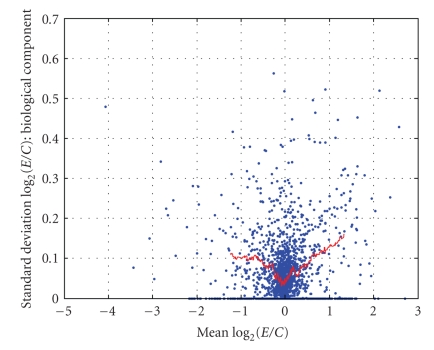
σ^b2 versus G^g.

**Figure 12:**
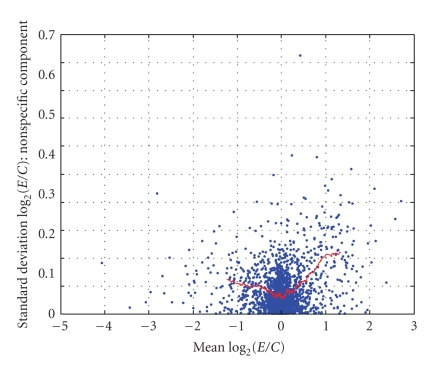
σ^ε2 versus G^g.

As described in Section 2.4, one degree-of-freedom estimates of the three variance components (σ^d2, σ^b2, and ${\hat{\sigma }}_{\varepsilon }^{2}$) were obtained for *each gene* via an analysis of variance (ANOVA) of the values of *R*. These summary statistics (G^g, σ^d2, σ^b2, and ${\hat{\sigma }}_{\varepsilon }^{2}$) were computed for *each gene* and are displayed in  Figures 9–12. Figure 9 displays the empirical cumulative distribution of estimated gene expression levels (${\hat{G}}_{g}$). For example, from this figure one can see that about 90% of the genes produced values of |G^g| that are less than one (or, exhibited less than a 2-fold change). Superimposed on the summary statistics in  Figures 10–12 are the “curves” that represent the “running 10%-trimmed mean” of the summary statistics (${\hat{\sigma }}_{d},{\hat{\sigma }}_{b},$ and ${\hat{\sigma }}_{\varepsilon }$) versus ${\hat{G}}_{g}.$

From Figure 10, one can conclude that the magnitude of the gene-specific effects associated with the dye status does not depend strongly on the level of $\hat{G}$ as the curve is nearly flat. Conversely,  Figures 11 and 12 show that the magnitudes of the “biological” and “nonspecific” sources of variation depend on the level of $\hat{G}$. As $\left|\hat{G}\right|$ increases, the magnitudes of the “biological” and “nonspecific” sources of variation increase. The asymmetry of the curves in  Figures 11 and 12 is interesting. The data indicate the biological (and nonspecific) variation of positively expressed genes exceeds that of negatively expressed genes. It should be noted that in some of our other experiments, we have noted much more variation across biological replicates and in the future we hope to identify and minimize the underlying sources of the variation across biological replicates.

### 3.4. Identification of Up-and Downregulated Genes

The ultimate objective of this study is to discover differences between the wild type and mutant strains in their response to their growth environment. The assessment whether a particular gene is upregulated or downregulated in the mutant (compared to the wild-type) is based on the test statistic Sg=G^g/σ^G^g(com), where σ^G^g(com)=max (σ^G^g,σ˜0) as discussed in  Sections 2.5 and 2.6. In the neighborhood around $\hat{G}=0$, we find that ${\overset{˜}{\sigma }}_{d}\approx 0.047,\;{\overset{˜}{\sigma }}_{b}\approx 0.048,\;{\overset{˜}{\sigma }}_{\varepsilon }\approx 0.067,$ and thus

(6) $${\overset{˜}{\sigma }}_{0}=\sqrt{\frac{{\overset{˜}{\sigma }}_{d}^{2}\left(0\right)}{2}+\frac{{\overset{˜}{\sigma }}_{b}^{2}\left(0\right)}{2}+\frac{{\overset{˜}{\sigma }}_{\varepsilon }^{2}\left(0\right)}{4}}=0.058.$$

**Figure 13:**
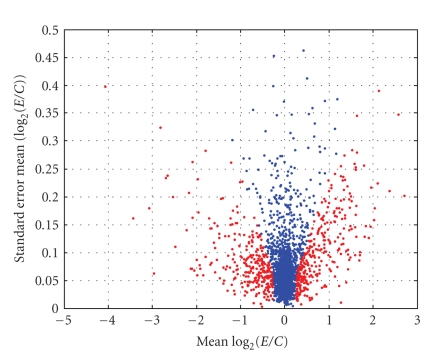
Significantly upregulated or downregulated genes (red): |*S*_*g*_| > 4.2.

Selected percentiles of the test statistic given in Table 2 were obtained by simulating expression data (assuming that *σ*_*d*_ = 0.047, *σ*_*b*_ = 0.048, and *σ*_*ε*_ = 0.067) as described in Section 2.6. An individual gene is declared as being significantly expressed (either up or down relative to the control) if |*S*_*g*_| > 4.2. This corresponds to a type-1 error of *α* = 0.00005, meaning that the likelihood of incorrectly declaring a specific gene (i.e., in fact nondifferentially expressive) as being significantly expressive is about 0.00005. Using the very conservative Bonferroni correction for the simultaneous inference of about 2000 genes, we have a type-1 error of about 0.10. Figure 13 illustrates the set of 629 genes that were declared as being significantly expressed relative to the control. Note that the significance analysis of microarrays (SAMs) procedure developed by Tusher et al. [19]) was not used in this example due to the fact that it is not possible to create a good resampling distribution with the very restricted number of possible permutations available with only 4 slides (see, e.g., [20]).

A similar process was used to assess the expression level associated with the 271 genes whose slide no. 2 measurements were anomalous (see  Figures 5 and 6). Again, we rely on the model *R*_*ij*_ = *G* + *B*_*i*_ + *D*_*j*_ + *ε*_*ij*_ with specified levels of the random effects given by *σ*_*d*_ = 0.047, *σ*_*b*_ = 0.048, and *σ*_*ε*_ = 0.067. Here, however, the simulation used to obtain the null distribution of the test statistic uses only three slides (since for these cases, results from three slides [rather than four slides] were used) and two biological samples. In this case, the test statistic is Sg*=G^g*/σ^G^g*(com), where

(7) G^g*=w1⋅R¯1⋅+w2⋅R21w1+w2,w1=1.5⋅σd2+.5⋅σε2+σb2, w2=1σd2+σε2+σb2,σ^G^g*(com)=max (σ^G^g,0.063),  σ^G^g2=1w^1+w^2,w^1=1.5⋅σ^w2+σ^b2, w^2=1σ^w2+σ^b2,σ^w2=∑i=12(R1i−R¯1⋅)2,σ^b2=max (0,(2⋅(R¯1⋅−R¯¯)2+(R21−R¯¯)2)−σ^w2(3−5/3)),R¯1⋅=.5⋅(R11+R12), R¯¯=13⋅(R11+R12+R21).

The estimates for *σ*_*w*_^2^ and *σ*_*b*_^2^ were obtained using methods for unbalanced data described in [17, page 72]. The second argument (0.063) in the definition for σ^G^g*(com) is the variance of G^g* obtained by assuming ${\overset{˜}{\sigma }}_{d}\left(0\right),\;{\overset{˜}{\sigma }}_{b}\left(0\right),$ and ${\tilde{\sigma }}_{\varepsilon }\left(0\right).$

From the simulation, we found approximate percentiles of the distribution of *S*_g_*. For example, the 0.000025 (0.999975) percentile was found to be about −3.95 (3.95). Thus, with a type-1 error of *α* = 0.00005, an individual gene is declared as being significantly expressed if |*S*_g_*| > 3.95. Of these 271 genes in question, 90 were deemed to be significantly expressed (43 positive and 47 negative).

Overall, across all 2408 genes considered (the 2137 genes represented on 4 slides plus the 271 genes represented on 3 slides), 719 genes were deemed to be significantly up- or downregulated. Tables 3 and 4 list the 15 genes that were the most upregulated and the 15 genes that were the most downregulated. The supplementary tables list all genes that were significantly up- or downregulated (See Tables 1 and 2 in the Supplementary Material available online at doi:10.1155/2009/950171). 

**Figure 14:**
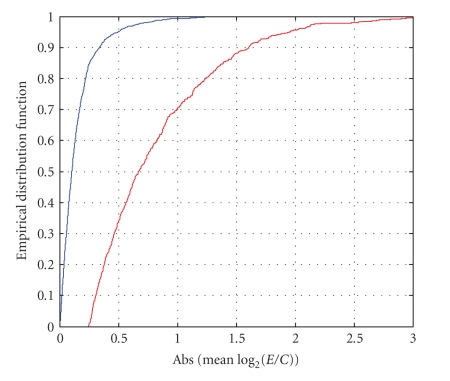
Cumulative distributions of |G^g| for selected (unselected) genes.

Figure 14 provides the cumulative distributions of |G^g| for both the selected and unselected genes. Based on the floor for σ^G^g(com) (${\overset{˜}{\sigma }}_{0}=0.058$ or ${\overset{˜}{\sigma }}_{0}=0.063$) and the selected threshold of 4.2 (or 3.95), the minimum level of |G^g|, such that gene is declared significant, is 4.2·0.058 ≈ 0.24 log_2_ relative expression units. About 1400 of the 2408 genes are associated with values of |G^g| less than 0.24 log_2_ relative expression units. Almost three quarters of the remaining genes (719 out of 993) were deemed to have been significantly expressed relative to the control. About 70% of the 719 significant genes exhibited less than a 2-fold change in intensity. About 35% of the significant genes exhibited less than a 1-fold change in intensity. Thus, we are able to identify large numbers of genes for which the treatment causes a small, but significantly different level in expression when compared to the control.

### 3.5. Biological Interpretations

One interesting biological outcome of these results is the extent to which changes in the phosphorus regulatory system seem to affect the gene expression of multiple genes beyond those strictly involved in phosphorus acquisition. This may be due to the many uses of phosphorus in the cell. It may also be due to the relatively small number of two-component regulatory systems in open ocean cyanobacteria, for example, only 5 histidine kinase sensors and 9 response regulators [11] and the possibility of substantial cross-talk among these systems. Inactivating one response regulator may affect this regulatory cross-talk. One unknown is whether the inactivation of SYNW0947 caused polar effects on nearby genes, especially the downstream *phoR* (SYNW0948) although this would still be part of changing the phosphorus regulatory system.

In addition, this statistical approach should allow for a much more robust identification of operons especially if gene expression in genes later in an operon are attenuated. The microarray results presented here suggest that several clusters of genes are potentially operons. For example, SYNW1016 and SYNW1017 were both significantly downregulated (see the supplementary tables). These are genes that are next to two other genes known to be involved in phosphate metabolism (SYNW1018 and SYNW1019). In addition a set of genes (SYNW0465-SYNW0470) were all highly upregulated and thus are a potential operon involved in phosphate metabolism. Interestingly, a third region probably comprising several operons (SYNW2477-2491) was also upregulated. These predictions merit further experimentation such as gene knockouts. As can be seen in Supplementary Figure 1 no spatial clustering of genes is apparent, suggesting that the operons detected are being found purely as a consequence of their place in regulatory networks affected by phosphate limitation.

We utilized the pathway analysis package DAVID (http://david.abcc.ncifcrf.gov/home.jsp) to examine the extent to which pathways, potentially involving multiple operons, are altered in the SYNW0947 mutant. The up- and downregulated genes from our analysis as well as using a simple 2-fold change statistic were mapped to KEGG pathways (see Supplementary Tables 1 and 2 where genes with simple 2-fold changes are shown in bold). We mapped 67 upregulated (of 360) genes to KEGG pathways while only 20 (of 100) genes were mapped using a 2-fold change. Our results demonstrated a much more convincing upregulation of the photosynthetic antenna proteins (9 genes) compared to the simpler analysis (5 genes). In addition new pathways involving mannose metabolism (SYNW0422, SYNW0423, SYNW0919) and other sugars were convincing upregulated in our analysis but were not seen with a 2-fold change statistic. We mapped 154 downregulated (of 337) genes to KEGG pathways compared to 40 (of 83) genes with a 2-fold change. Interestingly, we were able to map a larger fraction of downregulated genes to KEGG pathways. Again we saw a much more convincing downregulation of specific pathways. We found 28 ribosomal genes downregulated compared to 8 using a 2-fold statistic. Since these genes are likely to be coregulated, our results are biologically coherent. Similarly 15 photosynthesis genes were downregulated compared to 5 in the simpler analysis. Interestingly, the cells are downregulating core phycobilisome antenna proteins while upregulating rod proteins. This suggests that they are making fewer but larger light harvesting antenna complexes.

## 4. Conclusions

We have used a replicated dye-swap experiment with multiple spots per gene per array as a platform for comparing a regulatory mutant of *Synechococcus* sp. WH8102 with the wild type under defined growth conditions in artificial seawater. Our process for analyzing the experimental data includes utilizing simple graphical displays. These displays were used to assess spot quality, spatial variability within an array, array-to-array reproducibility, as well as other effects due to special causes (e.g., well plate). Quantitative analysis was based on the median expression level (within an array) of each gene. Following array normalization, a variance components analysis was used to partition the observed variability in expression level across replicate arrays. The level of variability introduced by dye swapping was found to be relatively small and independent of the apparent expression level. The variation in gene expression across biological replicates was found to be more significant and was found to be dependent on the apparent expression level. As only one strain was utilized, the biological significance of the data cannot be extended beyond the wild type strain used, but the statistical method developed with this model will allow greater sensitivity than was previously possible. The assessment of whether a particular gene is upregulated or downregulated was based on a test statistic that excludes genes that would otherwise be identified solely on the basis of a chance abnormally low level of variation across arrays. 

The null distribution of the test statistic was computed making a number of assumptions and by carefully constructing a simulation that mimicked the experiment and the observed sources of variation. A relatively large proportion of the genes were identified as being significantly upregulated or downregulated by the treatment, albeit with relatively small changes in the levels of expression. The ability to detect these small changes in the levels of expression (as small as about 0.25 log_2_ units) is a direct consequence of the replication within the array. 

## Supplementary Material

Supplemental tables show a comparison of the statistical method discussed here and a simple 2-fold change statistic. Up regulated and down regulated genes are found in supplemental Tables 1 and 2, respectively along with available annotation. In addition we have mapped the location of these genes on the genome of Synechococcus sp. WH8102 as seen in supplemental Figure 1 and show their frequent co-localization with regions of atypical % GC content.

## Figures and Tables

**Table 1:** Array assignment.

Slide	Cy3	Cy5
1	SYNW0947-sample no. 1	WH8102
2	WH8102	SYNW0947-sample no. 1
3	SYNW0947-sample no. 2	WH8102
4	WH8102	SYNW0947-sample no. 2

**Table 2:** Selected percentiles of *S*_*g*_ under assumption of no treatment effect based on 1 000 000 independent simulation realizations.

*α*/2	1 − *α*/2 percentile
.01	2.16
.001	2.95
.0001	3.6
.000025	4.2

**Table 3:** Statistically significant genes with highest level of upregulation. G^g: estimated relative expression level, *S*_*g*_: test statistic.

Gene ID	G^g	*S*_*g*_	Gene description
SYNW1555	2.72	13.47	Hypothetical
SYNW2478	2.58	7.42	Conserved hypothetical protein
SYNW2480	2.37	11.28	ABC transporter, ATP binding component, possibly zinc transport
SYNW0524	2.13	6.10	Conserved hypothetical protein
SYNW0424	2.13	5.46	Possible HMGL-like family protein
SYNW2481	2.10	9.36	Putative zinc transport system substrate-binding protein
SYNW1305	2.03	11.34	Hypothetical
SYNW0947	2.03	12.86	Two-component response regulator, phosphate
SYNW1463	2.02	32.03	Hypothetical
SYNW2479	1.96	9.04	ABC transporter component, possibly Zn transport
SYNW1654	1.95	13.34	Conserved hypothetical protein
SYNW2486	1.91	15.10	Putative cyanate ABC transporter
SYNW0454	1.84	12.43	Possible glycosyltransferase
SYNW1947	1.81	14.14	Conserved hypothetical protein
SYNW0456	1.79	7.00	Possible glycosyltransferase

**Table 4:** Significant genes with highest level of downregulation. G^g: estimated relative expression level, *S*_*g*_: test statistic.

Gene ID	${\hat{G}}_{g}$	*S*_*g*_	Gene description
SYNW2508	−4.07	−10.24	Molecular chaperone DnaK2, heat shock protein hsp70-2
SYNW0514	−3.44	−21.37	GroEL chaperonin
SYNW1503	−3.06	−17.05	Endopeptidase Clp ATP-binding chain B
SYNW1797	−2.96	−47.82	Putative iron ABC transporter, substrate binding protein
SYNW0513	−2.94	−41.756	GroES chaperonin
SYNW1278	−2.90	−19.209	Heat shock protein HtpG
SYNW2391	−2.81	−8.68	Putative alkaline phosphatase
SYNW1018	−2.69	−11.50	ABC transporter, substrate binding protein, phosphate
SYNW1798	−2.65	−11.14	Putative iron ABC transporter
SYNW1511	−2.58	−25.108	Conserved hypothetical
SYNW0938	−2.54	−12.69	Endopeptidase Clp ATP-binding chain C
SYNW2390	−2.48	−22.48	Putative alkaline phosphatase/5′ nucleotidase
SYNW0835	−2.22	−15.82	Probable oxidoreductase
SYNW1842	−2.17	−10.44	Apocytochrome f
SYNW0670	−2.14	−7.950	Conserved hypothetical protein
